# Analysis of factors influencing retinal thickness in chronic obstructive pulmonary disease and hypertension: a cross-sectional study in a community-dwelling middle-aged and elderly population

**DOI:** 10.3389/fmed.2026.1752515

**Published:** 2026-03-03

**Authors:** Donghui Yu, Chengyu Hu, Chengda Ren, Meijiang Zhu, Ruoyi Lin, Yan Wu, Zhongqi Wan, Tianyi Shen, Tingting Li, Wenting Cai, Jing Yu

**Affiliations:** 1 Department of Ophthalmology, Shanghai Pudong Hospital, Fudan University Pudong Medical Center, Shanghai, China; 2 Shanghai General Hospital, National Clinical Research Center for Eye Diseases, Shanghai Clinical Research Center for Eye Diseases, Shanghai Key Clinical Specialty, Shanghai Key Laboratory of Ocular Fundus Diseases, Shanghai Engineering Center for Visual Science and Photomedicine, Shanghai Engineering Center for Precise Diagnosis and Treatment of Eye Diseases, Shanghai, China; 3 Department of Ophthalmology, West China Hospital, Sichuan University, Chengdu, Sichuan, China; 4 Department of Ophthalmology, Changhai Hospital, Naval Medical University, Shanghai, China; 5 Department of Ophthalmology, Shanghai Tenth People’s Hospital, School of Medicine, Tongji University, Shanghai, China; 6 Department of Ophthalmology, Renji Hospital, School of Medicine, Shanghai Jiao Tong University, Shanghai, China; 7 Department of Ophthalmology, East Hospital, Tongji University School of Medicine, Shanghai, China

**Keywords:** chronic obstructive pulmonary disease, community population, diastolic blood pressure, hypertension, optical coherence tomography, retinal thickness, systolic blood pressure

## Abstract

**Background:**

Chronic obstructive pulmonary disease (COPD) and Hypertension are modifiable risk factors for premature mortality globally, with well-documented associations with retinal microvascular damage. Macular retinal thickness serves as a core quantitative indicator of macular structural integrity and ocular disease progression. However, existing studies primarily focus on retinal nerve layers in clinical populations, lacking investigations into full retinal thickness (RT) across all macular orientations in community-dwelling middle-aged and elderly individuals. This cross-sectional study aimed to measure RT in all macular regions using optical coherence tomography and identify hypertension as a key risk factor for retinal thickness abnormalities.

**Methods:**

Participants aged ≥40 years were recruited from community physical examinations between January and December 2020. After excluding ineligible participants, 1,522 participants (2,977 eyes) were included. Demographic data, lifestyle factors, comorbidities, and ocular indicators (blood pressure and RT via iVue OCT using the ETDRS grid) were collected. Statistical analyses included-independent samples t-test, chi-square test, linear regression, and logistics regression (*p* < 0.05 for significance).

**Results:**

The mean age of participants was 70.04 ± 5.740 years (55.0% female), with 49.8% diagnosed with hypertension and 8.2% diagnosed with COPD. Age was associated with retinal thickness thinning in all regions except the central area (*p* < 0.05). Stratification by hypertension status revealed no association between COPD and retinal thickness across any regions in the hypertensive subgroup, whereas in the non-hypertensive subgroup, COPD was significantly linked to thinning in the central, inner nasal, inferior outer, and outer nasal regions (*p* < 0.05). History of hypertension was a risk factor for thinning in the temporal inner and superior outer regions (OR = 1.249, 95%CI:1.030–1.514; OR = 1.325, 95%CI:1.092–1.608). Diastolic blood pressure was positively correlated with inner retinal thickness (*β* = 0.186–0.202, *p* < 0.01), while systolic blood pressure had no significant effect. In hypertensive participants on medication, DBP’s positive correlation was limited to the superior inner region.

**Conclusion:**

COPD and hypertension are independent risk factors for changes in retinal thickness, with heterogeneous effects across different retinal regions. DBP, but not SBP, independently correlates with inner retinal thickness, and antihypertensive therapy mitigates this effect to specific regions. These findings provide new insights for community ocular screening and retinal protection in hypertensive patients.

## 1 Introduction

Chronic obstructive pulmonary disease (COPD) is a slowly progressive chronic respiratory disorder, recognized as a leading global cause of mortality, with a prevalence of 10.3% among individuals aged 30–79 years worldwide (1), and contributing to 74.4 million disability-adjusted life years lost and 3.3 million deaths (2). COPD impacts ocular fundus structures via mechanisms including chronic hypoxia, systemic inflammation, and vascular dysfunction, resulting in alterations to retinal layer thickness. These changes are typically assessed using imaging modalities such as optical coherence tomography (OCT) (3).

Hypertension is globally recognized as the leading modifiable risk factor for premature mortality (4). Beyond its well-established role in cardiovascular, renal, and cerebrovascular diseases, hypertension induces retinal microvascular damage characterized by impaired vasomotor regulation and increased wall-to-lumen ratio (5). These alterations manifest as significant changes in multiple retinal nerve fiber layers in hypertensive patients compared with normotensive controls, ultimately affecting total retinal thickness (6).

Retinal thickness (RT) reflects the structural integrity, functional status, and pathological changes of the macular region, and serves as a core quantitative indicator for the progression of various ocular diseases. Central macular thickness (CMT) directly affects the density and function of cone cells, with a thickness of approximately 300 μm in this area (7, 8). CMT is not an isolated indicator; it can integratively reflect the effects of multiple influencing factors, and any abnormality in it may lead to decreased central visual acuity. Increased CMT thickness is often associated with local inflammation, vascular leakage, and fluid accumulation, and serves as an early marker for the active phase of vision-threatening diseases (9–11) such as diabetic retinopathy, retinal vein occlusion, and uveitis. In contrast, decreased thickness is often associated with high myopia and neurodegenerative diseases, such as optic neuropathy, late-stage dry age-related macular degeneration (AMD) (geographic atrophy), as well as some hereditary retinal diseases. CMT, measured by OCT, is an important parameter for diagnosing different types of retinal diseases (12–14).

The standardized segmentation of the fovea centralis of the macula and its surrounding areas is an important foundation for research on retinal structure and function. This segmentation was first mentioned in the 1985 Early Treatment Diabetic Retinopathy Study (ETDRS) (15) and is referred to as the ETDRS grid. It is divided into three concentric rings based on diameters of 1 mm, 3 mm, and 6 mm, and is further subdivided into four quadrants: superior, inferior, temporal, and nasal (16). The 1-mm foveal centralis region of the macula is a circular area with the fovea centralis as the center and a diameter of 1 mm, corresponding to an approximate range of 5° in the central visual field, and serves as the core region with the sharpest visual acuity. The 3-mm perifoveal region of the macular fovea is a ring-shaped area with the fovea as the center and a radius ranging from 1 mm to 3 mm. It corresponds to an approximate range of 10° in the central visual field and belongs to the parafovea. The 6-mm perifoveal region of the macular fovea is a ring-shaped area with the fovea as the center and a radius ranging from 3 mm to 6 mm, and corresponds to a range of 20° in the central visual field. The entire standardized segmentation with a 6-mm diameter fully covers the macular region. The retina in the fovea centralis of the macula and its surrounding superior, inferior, temporal, and nasal regions at different distances exhibits significant differences in structure, cell distribution, vascular supply, and function— differences that are closely associated with visual sensitivity and disease susceptibility (17). Understanding the influencing factors of retinal thickness in different macular regions provides a theoretical foundation for the risk assessment, staging, and treatment of macular-related diseases, and is of great significance for enhancing diagnostic and therapeutic efficiency and improving visual prognosis.

The thickness of different retinal nerve layers and the choroid in the population changes with increasing age and is influenced by multiple factors such as gender, lifestyle, disease status, and ocular factors (18, 19). However, existing studies mostly focus on different retinal nerve layers based on clinic-based patient studies (20, 21), and lack investigations on the full-thickness retinal thickness in all orientations of CMT among community-dwelling middle-aged and elderly populations.

This study is a cross-sectional study on community-dwelling middle-aged and elderly populations, accurately measuring retinal thickness in all orientations via OCT, while simultaneously collecting demographic data, lifestyle factors, comorbidities, and ocular indicators. It aims to establish age-specific reference ranges for retinal thickness in this population, to determine whether hypertension is a key risk factor for abnormal retinal thickness and to explore the impact of blood pressure control on RT. This study not only fills gaps in community-based research and provides appropriate assessment benchmarks for clinical practice, but also supports the development of ocular health screening strategies for community-dwelling middle-aged and elderly individuals and early warning of relevant ocular disease risks, contributing to the improvement of ocular health among middle-aged and elderly populations in the context of healthy aging.

## 2 Materials and methods

### 2.1 Study design

We recruited healthy community-based participants from a community in Shanghai ≥40 years of age undergoing physical examinations from January 1, 2020, to December 31, 2020. Out of the 1,653 subjects, those who were duplicated in data entry (*n* = 32), who lacked the data of COPD (*n* = 26), who lacked the data on hypertension (*n* = 56) and who with the history of diabetic retinopathy, retinal vein occlusion, hypertensive retinopathy (*n* = 17) were excluded. History of diabetic retinopathy, retinal vein occlusion and hypertensive retinopathy required prior diagnoses from hospitals graded II-A or higher. A total of 1,522 participants (2,977 eyes) were included in this study, and informed consent was acquired from each subject. This study adhered to the principles of The Declaration of Helsinki. Shanghai Tenth People’s Hospital approved this study (SHDSYY-2024-1902).

### 2.2 Sample size calculation

According to a case–control study on hypertension in China (22), the RT of participants with hypertension and healthy participants were 241.2 ± 20.6 μm and 250.7 ± 14.17 μm, respectively. Under the conditions of *α* = 0.05 (two-tailed), power of test 1-*β* = 90% (*β* = 0.1), design effect = 1.5, and a non-response rate of 10%, the total sample size estimated using the formula $n=2\times \frac{{\left(Z_{\alpha /2}+Z_{\beta }\right)}^{2}\times \sigma^{2}}{\delta^{2}}$ was 246 participants. The sample size in the present study met the requirements of the study design.

### 2.3 Clinical measurement

Gender, age, alcohol consumption status, history of COPD, history of coronary heart disease, history of hypertension, history of diabetes mellitus, history of hyperlipidemia, 30-min moderate-intensity exercise, weekly intake of fruits and vegetables, salty taste preference, and regular consumption of pickled food were included in this study. Inclusion of medical history of hypertension, coronary heart disease, diabetes mellitus, and COPD required prior diagnoses from hospitals graded II-A or higher. Daily blood pressure, including Diastolic blood pressure (DBP) and systolic blood pressure (SBP), was measured using a mercury sphygmomanometer. Before measurement, participants were asked whether they had consumed coffee, strong tea, or other similar beverages on the day of measurement, as well as whether they had eaten, consumed alcohol, exercised, or smoked prior to the test. If any of the aforementioned conditions applied, measurements were taken after the participants had rested for more than 30 min; in the absence of these conditions, measurements were taken after the participants had rested for more than 5 min.

Gender, age, alcohol consumption status history of coronary heart disease, history of diabetes mellitus, history of hyperlipidemia, 30-min moderate-intensity exercise, weekly intake of fruits and vegetables, salty taste preference, and regular consumption of pickled food were included as covariate.

**Figure 1:**
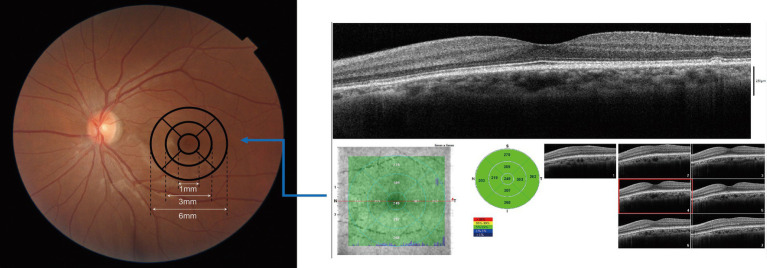
Schematic diagram of ETDRS grid. The macular fovea is divided into three concentric zones: a central zone (1 mm radius), an inner ring (3 mm radius), and an outer ring (6 mm radius).

OCT imaging was performed by experienced examiners using iVue OCT device (Optovue, United States). CMT was measured with a 6 × 6 mm combined scanning pattern centered on the macula. For average macular thickness analysis, the average thickness across all regions was used as the key parameter (Figure 1). Thickness values were also obtained for the central subfield (1 mm) and the parafoveal (1-3 mm)/ perifoveal (3-6 mm) sectors of the four quadrants (superior, nasal, inferior, and temporal) (Figure 1). For inclusion in the study, OCT images were required to meet the following criteria: acquired by experienced examiners for each participant, and free of artifacts, misalignment, or segmentation errors. RT was assessed by the iVue OCT system: measuring the distance between the inner border of the retinal pigment epithelium (RPE) and the inner limiting membrane (ILM). The device was equipped with a built-in Normative Database, which contained RT data from 500 healthy adults with balanced demographic features (covering diverse ethnicities, genders, and age groups). This database served to calculate the 95% confidence intervals (CI). Clinically, values exceeding the upper limit of the 95% CI were defined as retinal thickening, whereas those below the lower limit of the 95% CI were defined as retinal thinning (23). Prior to blood pressure measurement, participants were asked to take 10 min of quiet rest; subsequently, their blood pressure was measured twice using a mercury sphygmomanometer, and the average value was calculated to assess their daily blood pressure control status.

### 2.4 Data processing and statistical analysis

EpiData software (v.3.1) was utilized for data entry. SPSS software (v.30.0, SPSS Inc., United States) was utilized for statistical analysis. Data were visualized via GraphPad Prism (v.10.0, GraphPad Software Inc., United States). Independent samples t-test, chi-square test, linear regression model, and logistics regression model were used to analyze the effects of participants’ lifestyle and disease status on CMT in all orientations. *p*-value < 0.05 was considered statistically significant.

## 3 Results

### 3.1 General information

Among 1,522 participants, the mean age was 70.04 ± 5.740 years; 49.8% participants were aged 60–69 years, and there were 867 females (55.0%). There were few participants with history of COPD (8.2%), drinking (14.1%), hypertension (49.2%), coronary heart disease (14.5%), diabetes (25.3%), hyperlipidemia (24.4%), ocular surgery (10.7%), and cataract (43.6%). Regarding lifestyle habits, 45.9% of participants engaged in moderate-intensity exercise for more than 30 min over 7 times per week, while 32.9% consumed 1–1.5 kg of fruits and vegetables per week. Only 15.4% of participants perceived their taste as salty, and 15.9% regularly consumed pickled foods. Variables such as history of hypertension were associated with COPD. (Table 1).

**Table 1:** Characteristics, retinal thickness and blood pressure of the study population (1,522 participants, 2,977 eyes).

Characteristics	History of COPD		Participants (*n* = 1,522)		*p*
	No	Yes	*n*	%	
Age (years)	69.30 ± 5.721	71.81 ± 5.700	70.04 ± 5.740^#^		<0.001
<60	41	1	42	2.8	0.005*
60–69	713	40	753	49.5	
70–79	560	60	620	40.7	
≥80	93	14	107	7.0	
Sex					
Male	633	47	680	44.7	0.393
Female	774	68	842	55.3	
Drinking history					
No	1,206	103	1,309	86.0	0.252
Yes	201	12	213	14.0	
History of hypertension					
No	727	37	764	50.2	<0.001*
Yes	680	78	758	49.8	
History of hypertensive participants taking antihypertensive drugs (*n* = 758)					
No	49	5	54	7.1	0.796
Yes	631	73	704	92.5	
History of coronary heart disease					
No	1,253	45	1,298	85.3	<0.001*
Yes	154	70	224	14.7	
History of diabetes					
No	1,063	68	1,131	74.3	<0.001*
Yes	344	47	391	25.7	
History of hyperlipidemia					
No	1,087	60	1,147	75.4	<0.001*
Yes	320	55	375	24.6	
Moderate-intensity exercise over 30 min (times/week)					
0	227	28	255	16.8	0.017
1–2	314	34	348	22.9	
3–4	118	10	128	8.4	
5–6	91	4	95	6.2	
≥7	657	39	696	45.7	
Weekly intake of fruits and vegetables					
Almost not	35	4	39	2.6	0.940
<1 kg	346	46	392	25.8	
1–1.5 kg	474	21	495	32.5	
1.5–2 kg	212	20	232	15.2	
≥2 kg	340	24	364	23.9	
Salty taste preference					
No	1,195	90	1,285	84.4	0.058
Yes	212	25	237	15.6	
Regular consumption of pickled foods					
No	1,183	97	1,280	84.1	0.940
Yes	224	18	242	15.9	
Systolic blood pressure (mmHg)	135.91 ± 17.644	137.30 ± 17.945	136.02 ± 17.665 ^#^		0.420
<120 mmHg	234	17	251	16.5	0.928
120–139 mmHg	634	49	683	44.9	
140–159 mmHg	394	36	430	28.3	
160–179 mmHg	123	11	134	8.8	
≥180 mmHg	22	2	24	1.6	
Diastolic blood pressure (mmHg)	79.64 ± 10.553	77.69 ± 11.261	79.49 ± 10.617 ^#^		0.058
<80 mmHg	764	68	832	54.7	0.459
80–89 mmHg	406	35	441	29.0	
90–99 mmHg	188	9	197	12.9	
100–109 mmHg	43	3	46	3.2	
≥110 mmHg	6	0	6	0.4	
Total	1,407	115	1,522	100	

			Eyes (*n* = 2,977)		
			*n*	%	
Central thickness (μm)	259.15 ± 40.248	263.84 ± 39.009	264.00 ± 47.262 ^#^		0.092
Thinning	362	23	385	12.9	0.205
Normal	1,987	160	2,147	72.1	
Thickening	404	41	445	14.9	
Temporal inner thickness (μm)	296.28 ± 33.912	295.39 ± 27.983	296.57 ± 36.487 ^#^		0.704
Thinning	500	41	541	18.2	0.977
Normal	2,066	167	2,233	75.0	
Thickening	187	16	203	6.8	
Inferior inner thickness (μm)	301.60 ± 32.835	300.82 ± 28.579	302.36 ± 39.480 ^#^		0.730
Thinning	401	32	433	14.5	0.993
Normal	2,194	179	2,373	79.7	
Thickening	158	13	171	5.7	
Nasal inner thickness (μm)	308.66 ± 31.690	309.72 ± 31.952	309.67 ± 35.263 ^#^		0.632
Thinning	500	40	540	18.1	0.483
Normal	2,150	172	2,322	78.0	
Thickening	103	12	115	3.9	
Superior inner thickness (μm)	300.73 ± 31.684	300.44 ± 32.272	300.56 ± 33.994 ^#^		0.894
Thinning	2,058	158	619	20.8	0.242
Normal	568	51	2,216	74.6	
Thickening	127	15	142	4.8	
Temporal outer thickness (μm)	266.80 ± 33.964	264.65 ± 28.436	266.87 ± 36.585 ^#^		0.355
Thinning	442	45	487	16.4	0.156
Normal	2,065	155	2,220	74.6	
Thickening	246	24	270	9.1	
Inferior outer thickness (μm)	271.78 ± 30.525	272.01 ± 41.830	273.16 ± 33.675 ^#^		0.916
Thinning	268	36	304	10.2	0.005*
Normal	2,131	155	2,286	76.8	
Thickening	354	33	387	13.0	
Nasal outer thickness (μm)	291.75 ± 26.048	293.49 ± 49.075	292.26 ± 27.933 ^#^		0.380
Thinning	289	24	313	10.5	0.864
Normal	2,280	183	2,463	82.7	
Thickening	184	17	201	6.8	
Superior outer thickness (μm)	275.59 ± 28.379	277.50 ± 44.970	275.38 ± 30.212 ^#^		0.359
Thinning	507	42	549	18.4	0.930
Normal	2,030	163	2,193	73.7	
Thickening	216	19	235	7.9	

^#^Mean ± SD.

### 3.2 Participants’ retinal thickness status

**Figure 2:**
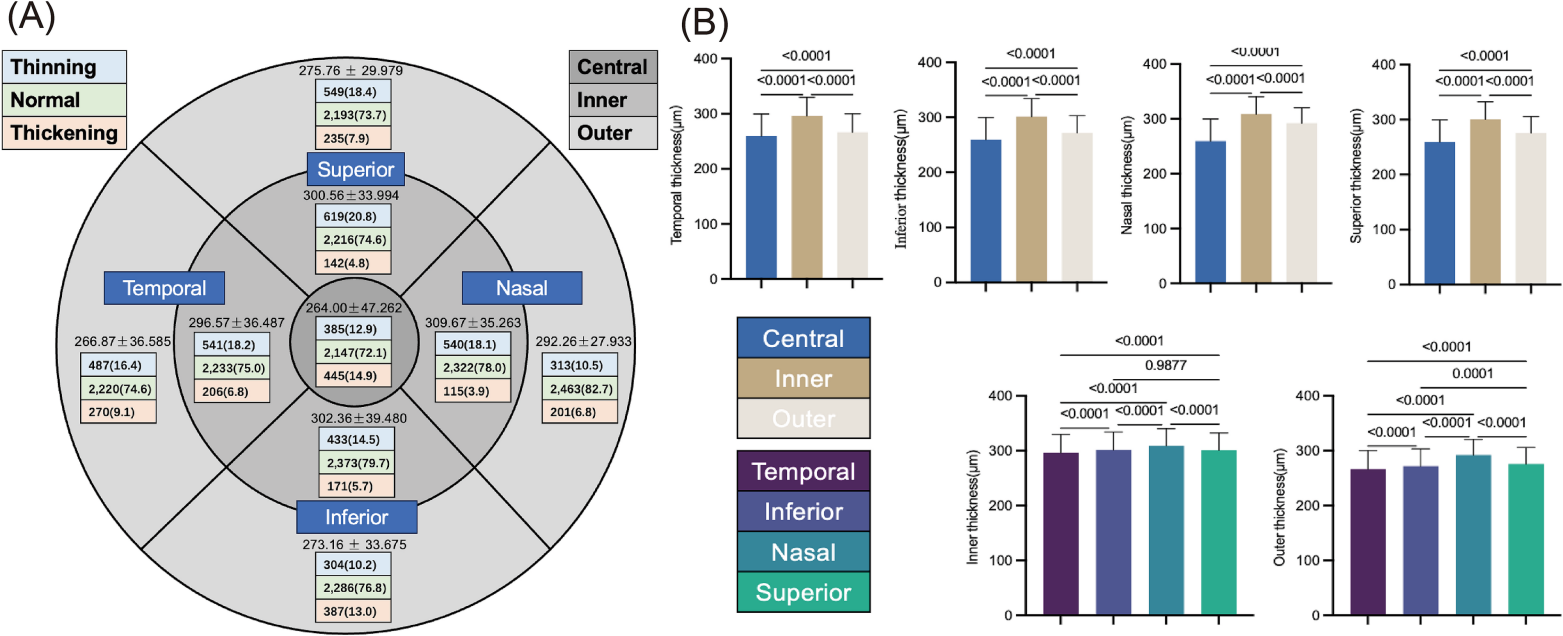
Retinal thickness in various regions. **(A)** Among the nine regions of the macular retinal area, the distinct RT (Mean±SD) of each region is presented, along with the number and proportion of eyes showing retinal thinning/normal/thickening thickness in each region; **(B)** *T*-test bar chart of RT in different regions, which examines the thickness differences of the central, inner, and outer retinal regions, as well as the thickness differences of the inner, inferior, nasal, and temporal sides of the retina.

Among 2,977 eyes, 385 eyes (12.9%) were thinned, 2,147 (72.1%) had normal thickness, and 445 (14.9%) were thickened in Central thickness (Table 1). The RT in the remaining quadrants, as well as the number (proportion) of thinning/normal/thickening eyes, are shown in Figure 2A. Whether in the temporal, nasal, superior, or inferior region, the central retina was the thinnest, followed by the outer circle, while the inner circle was the thickest. In the inner retina region, the nasal retinal region was thicker, the temporal was the thinnest, and the thickness of the superior and inferior was not significantly different. In the outer retinal region, the nasal retinal region was the thickest, followed by the superior and inferior, while the temporal was the thinnest (Figure 2B). The RT under different systolic/diastolic blood pressure categories were presented in Table 2.

**Table 2:** Retinal thickness in different retinal subfields among participants stratified by diastolic/systolic blood pressure.

Retinal region	Systolic blood pressure (mmHg)				
Mean ± SD (μm)	<120	120–139	140–159	160–179	≥180
Central	260.16 ± 42.994	257.65 ± 36.192	260.20 ± 41.712	262.55 ± 46.116	275.17 ± 48.611
Temporal Inner	295.95 ± 34.371	295.97 ± 30.264	296.02 ± 37.905	297.63 ± 30.708	301.19 ± 42.479
Inferior Inner	300.45 ± 33.025	300.48 ± 28.085	302.62 ± 37.305	303.54 ± 33.792	312.32 ± 42.764
Nasal Inner	308.24 ± 31.684	307.86 ± 28.842	308.88 ± 34.078	313.15 ± 37.603	311.72 ± 28.013
Superior Inner	300.59 ± 31.507	300.39 ± 29.085	300.47 ± 35.191	303.07 ± 32.462	301.77 ± 36.583
Temporal Outer	265.52 ± 32.433	267.63 ± 34.315	266.24 ± 34.186	264.26 ± 28.503	271.02 ± 39.428
Inferior Outer	270.64 ± 28.939	272.22 ± 32.622	272.16 ± 31.589	269.35 ± 28.453	279.00 ± 39.024
Nasal Outer	291.73 ± 25.557	291.78 ± 29.826	291.85 ± 27.578	292.54 ± 30.001	293.32 ± 22.653
Superior Outer	275.62 ± 28.381	276.41 ± 29.803	275.23 ± 31.560	274.52 ± 28.459	273.53 ± 28.996

	Diastolic blood pressure (mmHg)				
	<80	80–89	90–99	100–109	≥110
Central	258.73 ± 37.600	259.84 ± 40.815	257.78 ± 38.487	264.79 ± 37.886	357.45 ± 148.220
Temporal Inner	294.13 ± 31.768	297.90 ± 33.866	297.36 ± 36.526	299.83 ± 26.641	352.45 ± 84.550
Inferior Inner	299.45 ± 29.564	303.61 ± 34.493	300.93 ± 31.782	304.34 ± 20.334	387.09 ± 116.823
Nasal Inner	306.64 ± 30.280	311.19 ± 31.806	307.70 ± 31.702	312.58 ± 25.405	372.18 ± 107.427
Superior Inner	298.21 ± 31.368	302.99 ± 31.431	302.97 ± 32.953	302.89 ± 28.579	328.36 ± 56.906
Temporal Outer	265.76 ± 35.656	267.78 ± 29.856	265.29 ± 35.683	269.47 ± 19.899	313.27 ± 62.933
Inferior Outer	271.98 ± 31.914	272.69 ± 31.165	267.20 ± 30.285	273.28 ± 19.248	322.64 ± 70.647
Nasal Outer	291.43 ± 29.967	292.87 ± 27.114	289.70 ± 27.430	294.46 ± 17.394	326.91 ± 31.191
Superior Outer	275.00 ± 30.994	276.72 ± 28.723	274.97 ± 30.275	277.64 ± 22.414	300.00 ± 42.143

### 3.3 Univariate logistics regression revealed that a history of hypertension influenced retinal thickness in different regions

In the preceding descriptive analyses, we identified an association between history of hypertension and COPD. Accordingly, participants were stratified into hypertensive and non-hypertensive subgroups for separate statistical evaluations. In the non-hypertensive subgroup, retinal thickness exhibited statistically significant differences between individuals with and without COPD in select regions, whereas no significant differences were observed in any regions within the hypertensive subgroup. (Table 3).

**Table 3:** Retinal thickness in different retinal subfields among participants’ conditions of hypertension and COPD.

Retinal region	Hypertension(−)			Hypertension(+)		
COPD	No	Yes	P	No	Yes	P
Central	257.70 ± 37.211	273.33 ± 44.661	<0.001*	260.70 ± 43.231	259.35 ± 35.302	0.711
Temporal inner	296.44 ± 31.763	297.01 ± 31.951	0.881	296.10 ± 36.087	294.63 ± 25.972	0.625
Inferior inner	300.79 ± 30.716	307.61 ± 22.369	0.063	302.47 ± 34.957	297.60 ± 30.635	0.100
Nasal inner	308.37 ± 30.774	318.56 ± 37.787	0.007*	308.98 ± 32.653	305.53 ± 27.955	0.212
Superior inner	301.27 ± 31.588	303.03 ± 31.783	0.646	300.15 ± 31.788	299.21 ± 32.533	0.732
Temporal outer	267.74 ± 35.352	265.96 ± 35.518	0.677	265.80 ± 32.392	264.03 ± 24.49	0.512
Inferior outer	271.06 ± 30.041	278.42 ± 31.002	0.043*	272.54 ± 31.029	268.97 ± 45.862	0.205
Nasal outer	291.92 ± 27.099	299.44 ± 29.245	0.022*	291.57 ± 24.881	290.66 ± 55.943	0.722
Superior outer	277.41 ± 28.168	276.81 ± 31.227	0.860	273.64 ± 28.486	277.83 ± 50.277	0.120

**Figure 3:**
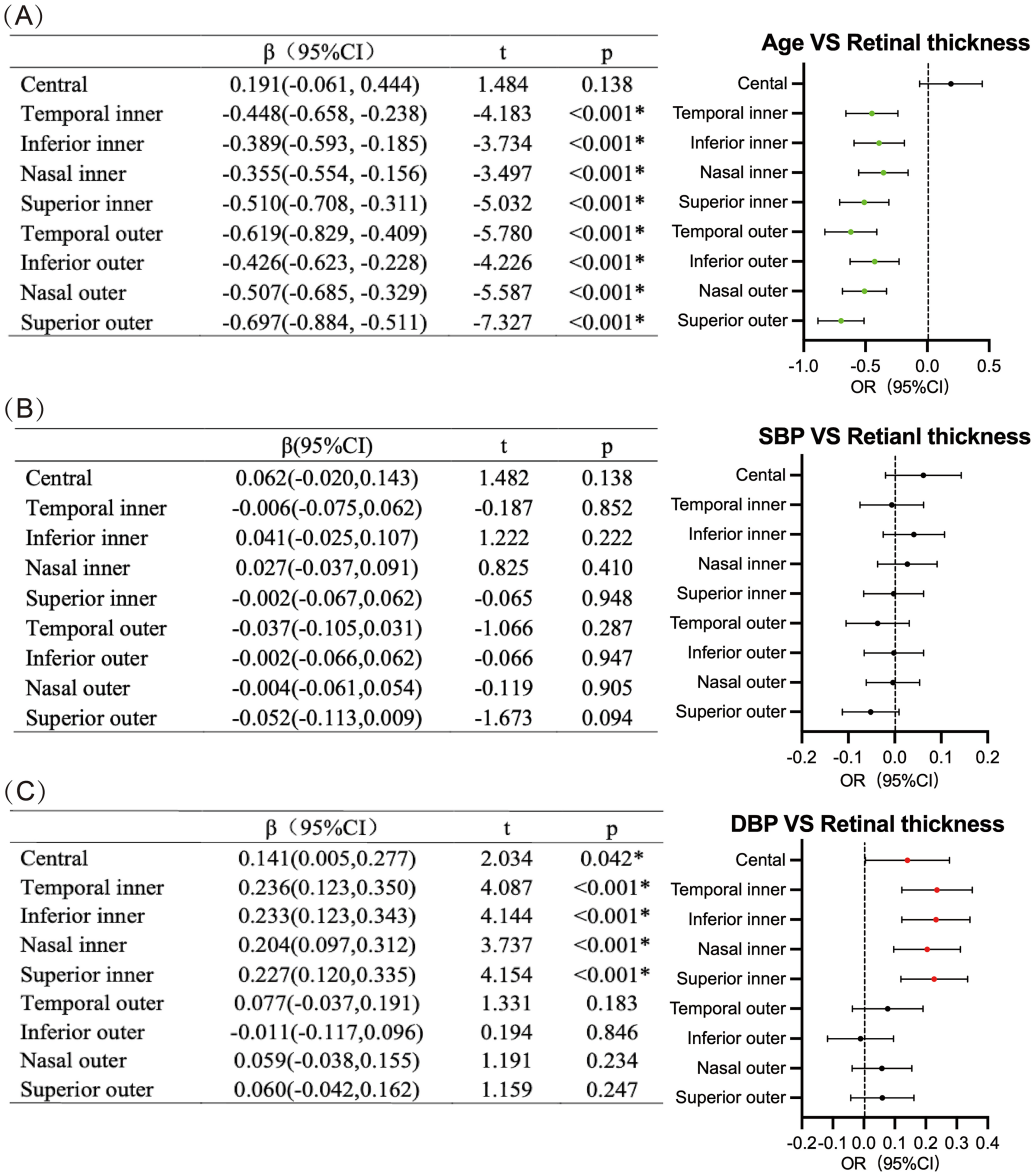
Results of univariate linear analysis among **(A)** age, **(B)** SBP, **(C)** DBP, and retinal thickness in different regions.

Subsequently, univariate linear analyses were performed separately to examine the relationships between age and RT, and between blood pressure and RT among the enrolled participants. These analyses revealed that RT in all regions except the central region thinned with increasing age (Figure 3A; Supplementary Figure S1A). In contrast, DBP was negatively correlated with RT in most of the retinal regions, while SBP had no statistical significance (Figure 3B; Supplementary Figures S1B,C). Meanwhile, univariate logistics regression analyses were performed to examine the association between retinal thickening/thinning and the following factors of the participants: gender, alcohol consumption history, history of COPD, history of hypertension, history of diabetes mellitus, history of coronary heart disease, history of hyperlipidemia,

30-min moderate-intensity physical activity, weekly vegetable and fruit intake, preference for salty taste and consumption of pickled foods. The other results were presented in Supplementary Figure S2. Both histories of hypertension and COPD influenced retinal thickness, with COPD predominantly affecting the non-hypertensive subgroup.

Univariate logistics regression analysis revealed that a history of hypertension was a potential risk factor for retinal thinning in the temporal inner region and superior outer region (Table 4). Therefore, a further univariate linear regression analysis was conducted on the subjects’ daily blood pressure, which demonstrated that DBP was.

**Table 4:** Univariate logistics regression analysis of history of hypertension and retinal thickness.

Retinal region	Thinning			Thickening		
Ref: No	*χ* ^2^	*p*	OR (95% CI)	*χ* ^2^	*p*	OR (95% CI)
Central	1.406	0.236	1.140 (0.918,1.417)	0.073	0.787	1.029 (0.839,1.261)
Temporal inner	5.556	0.018*	1.254 (1.039,1.514)	2.144	0.143	1.240 (0.930,1.654)
Inferior inner	0.253	0.615	1.054 (0.859,1.294)	1.311	0.252	1.199 (0.879,1.637)
Nasal inner	1.331	0.249	1.117 (0.926,1.347)	0.415	0.519	1.131 (0.778,1.645)
Superior inner	0.334	0.563	1.054 (0.882,1.260)	0.018	0.892	1.024 (0.729,1.437)
Temporal outer	1.606	0.205	1.135 (0.933,1.382)	0.000	0.983	1.003 (0.779,1.291)
Inferior outer	2.536	0.111	1.215 (0.956,1.545)	0.145	0.703	1.043 (0.841,1.294)
Nasal outer	2.888	0.089	1.227 (0.969,1.554)	0.015	0.902	0.982 (0.737,1.309)
Superior outer	6.672	0.010*	1.281 (1.062,1.546)	2.671	0.102	0.798 (0.608,1.046)

positively correlated with the thickness of the central and inner retinal regions, whereas SBP showed no statistical significance (Figures 3B,C; Supplementary Figures S1B,C).

### 3.4 Multivariable analysis revealed that the effect of diastolic blood pressure on retinal thickness was mitigated following the use of antihypertensive medications

**Figure 4:**
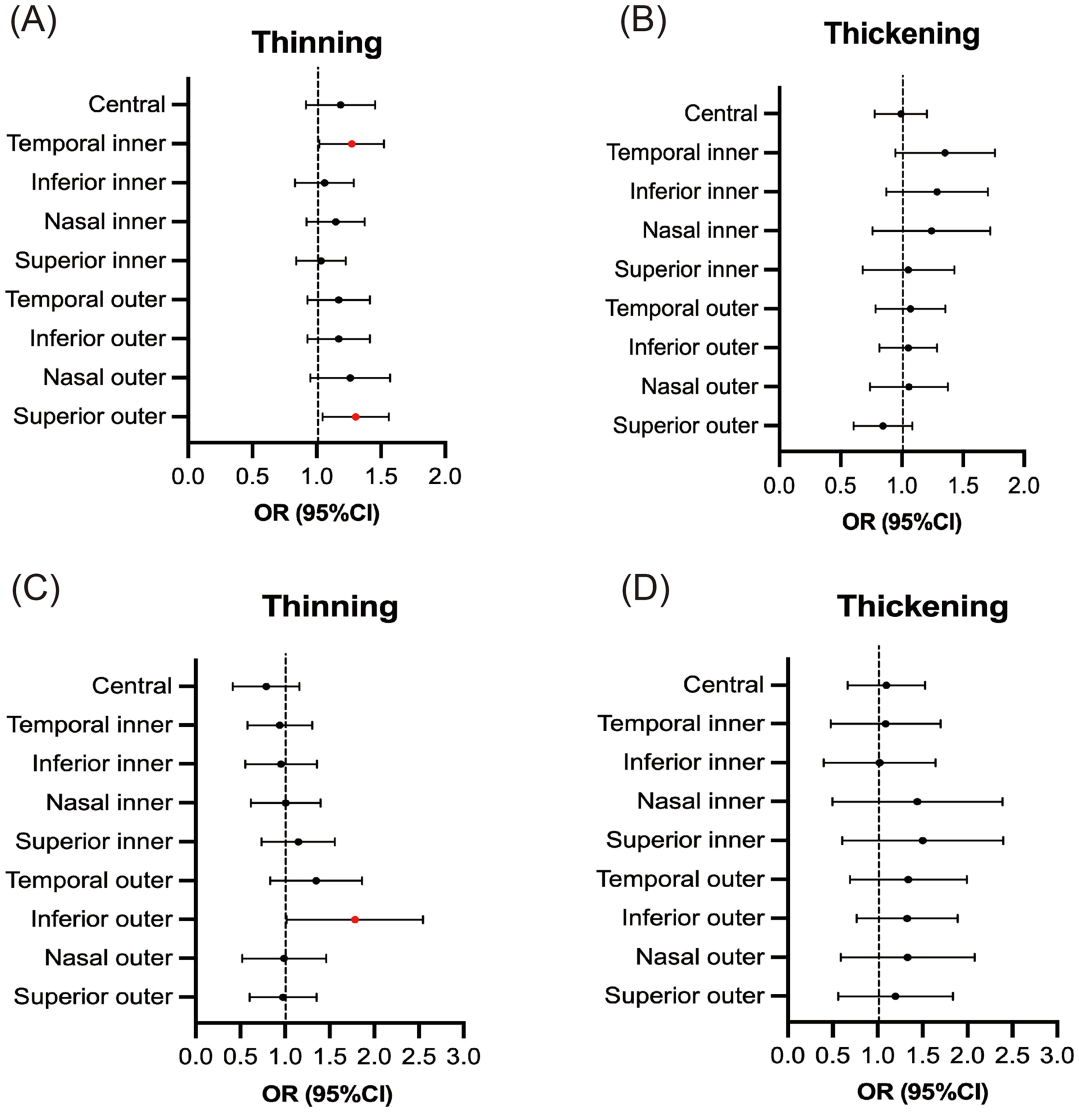
Forest map (reference: normal thickness): multivariate logistics analysis of history of hypertension/COPD and retinal thickness. **(A)** History of hypertension (thinning thickness); **(B)** History of hypertension (thickening thickness); **(C)** History of COPD (thinning thickness); **(D)** History of COPD (thickening thickness).

Finally, we performed a multivariable analysis, incorporating the aforementioned univariate variables (including history of hypertension and COPD) into a multivariable logistics regression model. After adjusting for other factors, the history of hypertension was identified as a risk factor for thinning of the temporal inner region and superior outer region, and history of COPD was a risk factor for thinning of inferior outer region (Table 5; Figure 4). Subsequently, we adjusted the model again, incorporating the participants’ SBP and DBP into the multivariable linear regression model separately. This analysis revealed that SBP had a limited effect on RT, whereas DBP was positively correlated with the thickness of the inner retinal region as DBP increased (Table 6). After adjusting the model once more, we focused on participants with a history of hypertension who were taking antihypertensive medications. In this subgroup, the effect of elevated DBP on the thickness of the inner retinal region was much more limited, with a positive correlation only observed in the superior inner region. Meanwhile, SBP still had a limited effect on RT, showing a negative correlation solely in the temporal outer region (Table 7).

**Table 5:** Multivariable logistics regression analysis of retinal thickness.

Reference no	Thinning (ref: normal)				Thickening (ref: normal)			
	*β*	*χ* ^2^	*p*	OR (95% CI)	*β*	*χ* ^2^	*p*	OR (95% CI)
Central thickness								
History of hypertension	0.153	1.733	0.188	1.165 (0.928,1.464)	−0.023	0.043	0.835	0.977 (0.788,1.213)
History of COPD	−0.316	1.610	0.205	0.729 (0.447,1.188)	0.038	0.034	0.853	1.039 (0.695,1.553)
Temporal inner thickness								
History of hypertension	0.228	5.058	0.025*	1.256 (1.030,1.533)	0.273	3.137	0.077	1.314 (0.971,1.779)
History of COPD	−0.112	0.314	0.575	0.894 (0.604,1.323)	−0.024	0.006	0.936	0.976 (0.544,1.751)
Inferior inner thickness								
History of hypertension	0.043	0.154	0.695	1.044 (0.841,1.296)	0.218	1.722	0.189	1.244 (0.898,1.724)
History of COPD	−0.107	0.236	0.627	0.899 (0.584,1.382)	−0.110	0.113	0.737	0.896 (0.473,1.698)
Nasal inner thickness								
History of hypertension	0.125	1.528	0.216	1.133 (0.930,1.381)	0.166	0.681	0.409	1.181 (0.796,1.751)
History of COPD	−0.044	0.048	0.826	0.957 (0.646,1.418)	0.213	0.361	0.548	1.237 (0.618,2.476)
Superior inner thickness								
History of hypertension	0.022	0.051	0.821	1.022 (0.847,1.233)	0.010	0.003	0.955	1.010 (0.705,1.448)
History of COPD	0.095	0.263	0.608	1.100 (0.765,1.582)	0.281	0.779	0.377	1.325 (0.709,2.475)
Temporal outer thickness								
History of hypertension	0.143	1.826	0.177	1.154 (0.938,1.421)	0.046	0.114	0.736	1.047 (0.801,1.369)
History of COPD	0.249	1.574	0.210	1.282 (0.869,1.892)	0.212	0.688	0.407	1.236 (0.749,2.038)
Inferior outer thickness								
History of hypertension	0.158	1.494	0.222	1.171 (0.909,1.510)	0.034	0.089	0.766	1.035 (0.825,1.298)
History of COPD	0.515	5.305	0.021*	1.673 (1.080,2.593)	0.223	1.019	0.313	1.250 (0.811,1.926)
Nasal outer thickness								
History of hypertension	0.211	2.784	0.095	1.235 (0.964,1.582)	0.027	0.030	0.862	1.027 (0.759,1.390)
History of COPD	−0.090	0.128	0.720	0.914 (0.560,1.493)	0.179	0.366	0.545	1.196 (0.670,2.137)
Superior outer thickness								
History of hypertension	0.251	6.153	0.013*	1.286 (1.054,1.568)	−0.195	1.775	0.183	0.823 (0.618,1.096)
History of COPD	−0.075	0.140	0.708	0.928 (0.628,1.372)	0.083	0.086	0.769	1.086 (0.625,1.887)

*All the above results were adjusted for the following variables: gender, age, history of alcohol consumption, history of coronary heart disease, history of diabetes mellitus, history of hyperlipidemia, 30 min of moderate-intensity exercise per week, weekly intake of vegetables and fruits, preference for salty food, and frequency of pickled food consumption.

**Table 6:** Multivariable linear analysis of diastolic blood pressure/ systolic blood pressure and retinal thickness.

SBP	*β* (95% CI)	*t*	*p*
Central	0.059 (−0.024,0.142)	1.390	0.165
Temporal inner	0.019 (−0.050,0.088)	0.540	0.589
Inferior inner	0.061 (−0.006,0.128)	1.788	0.074
Nasal inner	0.044 (−0.022,0.109)	1.308	0.191
Superior inner	0.023 (−0.042,0.088)	0.694	0.488
Temporal outer	−0.008 (−0.077,0.061)	−0.230	0.818
Inferior outer	0.013 (−0.052,0.079)	0.401	0.688
Nasal outer	0.016 (−0.042,0.075)	0.550	0.582
Superior outer	−0.023 (−0.085,0.038)	−0.740	0.460
DBP			
Central	0.128 (−0.010,0.265)	1.815	0.070
Temporal inner	0.194 (0.080,0.308)	3.332	<0.001*
Inferior inner	0.202 (0.091,0.313)	3.571	<0.001*
Nasal inner	0.169 (0.061,0.277)	3.057	0.002*
Superior inner	0.187 (0.078,0.295)	3.384	<0.001*
Temporal outer	0.028 (−0.087,0.142)	0.476	0.634
Inferior outer	−0.025 (−0.133,0.083)	−0.452	0.652
Nasal outer	0.036 (−0.061,0.133)	0.724	0.469
Superior outer	0.034 (−0.068,0.136)	0.656	0.512

**All the above results were adjusted for the following variables: gender, age, history of COPD, history of alcohol consumption, history of coronary heart disease, history of diabetes mellitus, history of hyperlipidemia, 30 min of moderate-intensity exercise per week, weekly intake of vegetables and fruits, preference for salty food, and frequency of pickled food consumption.

**Table 7:** Multivariablelinear analysis of diastolic blood pressure/ systolic blood pressure and retinal thickness among hypertensive participants taking antihypertensive drugs.

SBP	*β* (95% CI)	*t*	*p*
Central	0.021 (−0.126,0.168)	0.282	0.778
Temporal inner	−0.102 (−0.222,0.017)	−1.677	0.094
Inferior inner	−0.002 (−0.123,0.118)	−0.040	0.968
Nasal inner	−0.037 (−0.151,0.076)	−0.645	0.519
Superior inner	−0.037 (−0.151,0.076)	−0.645	0.519
Temporal outer	−0.135 (−0.239,-0.030)	−2.529	0.012*
Inferior outer	−0.056 (−0.164,0.051)	−1.027	0.305
Nasal outer	−0.054 (−0.140,0.033)	−1.221	0.222
Superior outer	−0.082 (−0.180,0.015)	−1.655	0.098
DBP			
Central	0.038 (−0.219,0.295)	0.287	0.774
Temporal inner	0.068 (−0.141,0.278)	0.641	0.522
Inferior inner	0.148 (−0.063,0.358)	1.377	0.169
Nasal inner	0.197 (−0.002,0.396)	1.938	0.053
Superior inner	0.209 (0.019,0.398)	2.162	0.031*
Temporal outer	−0.040 (−0.224,0.143)	−0.429	0.668
Inferior outer	−0.166 (−0.355,0.022)	−1.734	0.083
Nasal outer	0.047 (−0.104,0.198)	0.611	0.541
Superior outer	0.089 (−0.082,0.260)	1.021	0.307

**All the above results were adjusted for the following variables: gender, age, history of COPD, history of alcohol consumption, history of coronary heart disease, history of diabetes mellitus, history of hyperlipidemia, 30 min of moderate-intensity exercise per week, weekly intake of vegetables and fruits, preference for salty food, and frequency of pickled food consumption.

## 4 Discussion

In recent years, studies have found that as an extension of the central nervous system, the retinal thickness changes could reflect the health status of the nervous system and the whole body (24). RT is not an isolated indicator. It integrates and reflects the combined effects of multiple influencing factors. For instance, vascular factors (such as hypertensive retinopathy) cause leakage or ischemia; inflammatory factors damage the blood-retinal barrier and induce fluid accumulation; neurodegenerative factors (such as glaucoma and Alzheimer’s disease) lead to the loss of the ganglion cell layer; and age-related and genetic factors also exert an impact on RT within a certain range. Investigating the influencing factors of RT essentially involves exploring the ultimate manifestation of the interactions among the aforementioned multiple systems, including vascular, neurological, inflammatory, and genetic systems (24–27).

Consistent with previous studies (28), we found that increasing age is associated with retinal thinning. This phenomenon is primarily attributed to age-related neuronal loss, gliosis, and degeneration of the retinal pigment epithelium (RPE) layer. These processes lead to cell apoptosis and structural atrophy, which in turn accelerate local microcirculatory disturbances and impaired metabolic function. Among these regions, the central region—being a macular area with high neuronal density—is more susceptible to accumulated oxidative stress, resulting in significant thinning.

Our findings indicate that in multivariable logistics regression model history of hypertension serves as an independent risk factor for retinal thinning in the inner temporal and superior outer regions, while COPD independently contributes to thinning in the inferior outer region. Notably, stratification by hypertension status revealed no association between COPD and retinal thickness across any regions in the hypertensive subgroup, whereas in the non-hypertensive subgroup, COPD was significantly linked to thinning in the central, inner nasal, inferior outer, and outer nasal regions (chi-square analysis). These observations underscore a potential interaction wherein hypertension may modulate COPD’s ocular effects. Focusing on COPD’s influence on retinal thickness, our results align with emerging evidence that COPD was associated with reduced retinal nerve fiber layer (RNFL) thickness, correlating with disease severity and hypoxemia levels, as detected via OCT. (3, 29) These changes likely stem from hypoxia-induced neuronal damage and vascular remodeling, disproportionately affecting outer retinal zones where oxygen demand is high (30). Furthermore, COPD’s microvascular retinopathy persists even after adjusting for comorbidities like hypertension, suggesting an intrinsic pathological pathway.

The stratified analysis highlights a novel interaction: COPD’s impact on retinal thickness appears confined to non-hypertensive individuals, potentially due to hypertension’s dominant vascular pathology overshadows COPD-related effects in comorbid cases. This could reflect additive or synergistic mechanisms in isolated COPD, where unmasked hypoxemia drives regional thinning, versus a ceiling effect in hypertensive patients with pre-existing endothelial impairment (31, 32). COPD and hypertension may synergistically affect retinal thickness through chronic hypoxia, systemic inflammation, and vascular endothelial dysfunction, collectively exacerbating microcirculatory impairment and neurodegeneration in specific retinal regions (20, 29).

Beyond the above findings, this study focused on the impact of hypertension on RT. After adjusting for lifestyle and medical history factors—including gender, age, alcohol consumption history, history of coronary heart disease, history of diabetes, history of hyperlipidemia, 30-min moderate-intensity exercise per week, weekly intake of fruits and vegetables, preference for salty food, and regular consumption of pickled foods—we found that participants with a previous history of hypertension were prone to thinning in the temporal inner region and superior outer region. Furthermore, based on further measurements of participants’ daily blood pressure, DBP was positively correlated with the thickness of the inner retinal region, while its impact on other retinal regions was not statistically significant. In contrast, SBP had no statistically significant effect on any retinal region. This indicated that a previous history of hypertension only induces changes in RT in specific regions. In contrast, daily blood pressure control—particularly of DBP rather than SBP—was associated with RT, especially in the inner retinal region. This provides guidance for retinal protection in patients with hypertension.

Patients with hypertension exhibit relatively lower vascular density (33). Retinal microvascular stenosis may increase microcirculatory resistance, cause impaired autoregulation of retinal blood flow, reduce microvascular blood flow, and easily lead to insufficient perfusion of the optic nerve, thereby resulting in retinal ischemia (34). Meanwhile, chronic elevated blood pressure induces retinal microvascular remodeling, endothelial damage, and local ischemia-hypoxia. These processes accelerate the degenerative changes of the outer plexiform layer, photoreceptor layer, and RPE, leading to cell apoptosis and structural atrophy, which further thin the retina. Retinal thinning may in turn reduce the oxygen consumption and blood flow of retinal blood vessels, triggering a vicious cycle of retinal structural atrophy and functional impairment (22). Previous studies have also found that long-term hypertension is associated with reduced retinal blood flow and thinning of the ganglion cell-inner plexiform layer (GC-IPL), RNFL, and other layers (33, 35, 36). This is consistent with our finding that participants with a history of hypertension showed thinning in the temporal inner region and superior outer region.

Our study found that a history of hypertension is a potential risk factor for thinning of the temporal inner and superior outer retina. Further assessment of daily blood pressure revealed that, compared with the limited effect of SBP on RT, DBP exerts an impact on the thickness of the inner retina across the entire macular region. DBP reflects the sustained pressure load on vascular walls during diastole and regulates continuous perfusion levels and basal vascular tone (37). The central retinal artery and its branches are classified as small-to-medium arteries and capillaries, which have thin vessel walls and weak regulatory capacity. In contrast, choroidal vessels belong to a “large vessel-sinusoidal capillary” system, characterized by thicker walls, larger diameters, and regulation by the autonomic nervous system and local metabolic factors. These features enable choroidal vessels to rapidly buffer blood pressure fluctuations through self-constriction and dilation (38, 39). Among different retinal regions based on the ETDRS 9-field grid, the inner retina relies on blood supply from the central retinal artery. Its vascular structure is more susceptible to systemic blood pressure fluctuations than the outer retina (which depends on choroidal perfusion) and the avascular foveal center. The core cells in the foveal center are cone cells, whose metabolic needs primarily rely on oxygen and nutrients supplied by the choroid. The key cell layers in the inner retina include the nerve fiber layer, inner nuclear layer, and Müller glial cells, while those in the outer retina consist of the photoreceptor layer and RPE layer. Therefore, the cell layers of the foveal center and outer retina lack the conditions to be affected by DBP (40, 41). The nerve fiber layer and ganglion cell layer in the inner retina are sensitive to hypoxia and oxidative stress. Elevated DBP can increase vascular permeability, leading to leakage of plasma components into the interstitial space and subsequent thickening. Meanwhile, retinal arteriolosclerosis reduces blood perfusion, prompting local compensatory thickening to maintain function. Sustained mechanical stress and ischemia-hypoxia further stimulate the proliferation of glial cells (e.g., Müller cells), which exacerbates inner retinal thickening (42, 43). SBP primarily reflects peak arterial pressure, and its elevation lasts only 0.2–0.3 s. This short-term peak cannot trigger cellular compensatory responses, resulting in a minimal independent contribution to chronic retinal structural changes. Thus, the effect of SBP is often masked by factors such as age, lifestyle, and comorbidities, leading to no significant linear association (44, 45). Both the central retinal artery and choroidal vessels possess myogenic autoregulatory capacity. In addition to myogenic regulation, choroidal vessels are also controlled by the autonomic nervous system and local metabolic factors. When SBP increases, the smooth muscles of the supplying arteries rapidly adjust blood flow through relaxation or contraction, thereby reducing the actual intravascular perfusion pressure. This mechanism ensures stable perfusion and prevents vascular damage or edema caused by sudden increases in blood flow (44, 46).

However, in other studies, elevated DBP often exhibits a negative correlation with the thickness of most retinal neural layers, including the RNFL, GCL, and outer segments of photoreceptors (POS). This is likely due to vasospasm and neuronal loss under high pressure dominating degenerative changes. In contrast, DBP shows a positive correlation with the thickness of the inner nuclear layer (INL), possibly resulting from inflammatory thickening of bipolar cells and glial cells in the INL that serves as a protective response (47). The positive correlation observed in this study may reflect the comprehensive effect on total RT, in which the positive contribution of the INL predominates in the multivariable model, masking the negative effects of other layers. This discrepancy may be attributed to differences in measurement methods between OCT-based measurements of total thickness in segmented regions and neural layer-specific analysis, which requires further verification.

In participants with a history of hypertension who were taking antihypertensive medications, the impact of blood pressure on inner retinal thickness was generally significantly limited. Antihypertensive medications protect the retina from chronic high-pressure damage by reducing overall blood pressure load, improving vascular compliance, and mitigating oxidative stress. The superior inner region has higher vascular density and blood flow, making it more sensitive to short-term variability in DBP (48). Even if medications control average blood pressure, residual fluctuations may still affect the microcirculation in this region, leading to tissue thickening. Compared with other regions, the vascular walls in the superior inner region may be more prone to remodeling (49, 50), which manifests as a positive correlation rather than a negative one in treated patients. The retinal vascular distribution in the temporal outer region is relatively sparse, making it more vulnerable to high pressure-induced local ischemia or oxidative stress. Chronic atrophy of nerve fibers results in reduced thickness. Although antihypertensive medications reduce overall blood pressure load, they cannot completely eliminate the residual effects of chronic hypertension on local microcirculation, ultimately leading to a negative correlation between blood pressure and thickness in this region (44). Elevated DBP showed a positive correlation only in the superior inner region, with no significant impact observed in other regions. This indicates that the potential negative effects of elevated DBP are attenuated and confined to specific local areas. Similarly, SBP exhibited a negative correlation only in the temporal outer region, with no obvious association detected in other sites. Overall, these findings highlight the protective role of antihypertensive therapy in alleviating the impact of blood pressure on retinal structure, reducing the global effect of blood pressure changes on the retina to specific anatomical regions.

This study also has several limitations. First, the study sample was derived from a single center and employed a cross-sectional design. Consequently, it can only identify associations rather than establish causality. Although we adjusted for multiple demographic and clinical confounders in the analysis, the single-center design may still limit the direct generalizability of our conclusions to other regions or broader populations due to factors such as regional demographic composition, genetic background, environmental exposures, and healthcare practices. Future multi-center, large-sample prospective studies are needed to further validate and extend our findings. Second, a notable limitation of this study is the lack of detailed information on the class, dosage, and duration of antihypertensive medications. Growing evidence suggests that different classes of antihypertensive agents (e.g., calcium channel blockers, ACE inhibitors/angiotensin receptor blockers, *β*-blockers) exert distinct effects on retinal vessel diameter, blood flow, and retinal nerve fiber layer thickness. Future studies will systematically collect these medication details and conduct subgroup analyses to elucidate the specific impact of different antihypertensive drug classes and dosages on retinal structure and function. This would provide more precise ophthalmological evidence for personalized management of hypertensive patients. Tirdly, this study has mentioned limitations including single-center setting, cross-sectional design, and lack of detailed information on antihypertensive medications. It is recommended to further supplement: The study did not include COPD severity classification (e.g., GOLD grade) and pulmonary function indicators (e.g., FEV1/FVC), making it impossible to clarify the dose–response relationship between COPD progression and retinal thickness.

This study holds great significance. It analyzed the differential effects of a history of hypertension, SBP, and DBP on the thickness of the nine retinal regions using multivariable linear and multivariable logistic regression analyses. Given the limited availability of ophthalmic examination equipment in settings such as community screenings, and considering that previous studies have focused on the impact of hypertension on individual retinal layers, this study centered on the relationship between total RT and hypertension. It revealed the independent positive correlation effect of DBP and further suggested that the use of antihypertensive medications can restrict this positive correlation. This finding not only deepens the understanding of the microscopic ocular effects of hypertension but also highlights the value of refined blood pressure assessment in preventing eye diseases. Furthermore, it provides new insights for community screening and the prevention of ocular disorders.

## 5 Conclusion

The study found that a history of hypertension is a potential risk factor for thinning of the temporal inner and superior outer retina. COPD and hypertension are independent risk factors for changes in retinal thickness, with heterogeneous effects across different retinal regions. Further evaluation of daily blood pressure levels revealed that, compared with the limited impact of SBP on RT, DBP exhibits a positive correlation with the thickness of the inner region of the entire macular region. In the subgroup of participants with a history of hypertension who were taking antihypertensive medications, the impact of DBP on inner retinal thickness was only observed in its positive correlation with the thickness of the inferior inner region. This indicates that elevated DBP affects the thickness of the inner retinal region, but this potential positive effect is attenuated after the use of antihypertensive medications and confined to specific local regions. It provides new insights into the impact of hypertension prevention and management on the retinal and optic nerve in the eye.

## Data Availability

The raw data supporting the conclusions of this article will be made available by the authors, without undue reservation.
