# Supplementary material for: Analysis of factors influencing retinal thickness in chronic obstructive pulmonary disease and hypertension: a cross-sectional study in a community-dwelling middle-aged and elderly population
Source: Front Med (Lausanne). 2026 Mar 3;13:1752515. doi: 10.3389/fmed.2026.1752515 (PMC12994154; doi:10.3389/fmed.2026.1752515)
Supplement: Supplementary file 1 [file Data_Sheet_1.PDF]

Univariate Analysis Results

Univariate Linear Analysis of Age and Retinal Thickness

	β(95%CI)	t	p
Central	0.191(-0.061, 0.444)	1.484	0.138
Temporal inner	-0.448(-0.658, -0.238)	-4.183	<0.001*
Inferior inner	-0.389(-0.593, -0.185)	-3.734	<0.001*
Nasal inner	-0.355(-0.554, -0.156)	-3.497	<0.001*
Superior inner	-0.510(-0.708, -0.311)	-5.032	<0.001*
Temporal outer	-0.619(-0.829, -0.409)	-5.780	<0.001*
Inferior outer	-0.426(-0.623, -0.228)	-4.226	<0.001*
Nasal outer	-0.507(-0.685, -0.329)	-5.587	<0.001*
Superior outer	-0.697(-0.884, -0.511)	-7.327	<0.001*

Univariate Linear Analysis of Diastolic Blood Pressure and Retinal Thickness

	β(95%CI)	t	p
Central	0.141(0.005,0.277)	2.034	0.042*
Temporal inner	0.236(0.123,0.350)	4.087	<0.001*
Inferior inner	0.233(0.123,0.343)	4.144	<0.001*
Nasal inner	0.204(0.097,0.312)	3.737	<0.001*
Superior inner	0.227(0.120,0.335)	4.154	<0.001*
Temporal outer	0.077(-0.037,0.191)	1.331	0.183
Inferior outer	-0.011(-0.117,0.096)	0.194	0.846

Nasal outer	0.059(-0.038,0.155)	1.191	0.234
Superior outer	0.060(-0.042,0.162)	1.159	0.247

Univariate Linear Analysis of Systolic Blood Pressure and Retinal Thickness

	β(95%CI)	t	p
Central	0.062(-0.020,0.143)	1.482	0.138
Temporal inner	-0.006(-0.075,0.062)	-0.187	0.852
Inferior inner	0.041(-0.025,0.107)	1.222	0.222
Nasal inner	0.027(-0.037,0.091)	0.825	0.410
Superior inner	-0.002(-0.067,0.062)	-0.065	0.948
Temporal outer	-0.037(-0.105,0.031)	-1.066	0.287
Inferior outer	-0.002(-0.066,0.062)	-0.066	0.947
Nasal outer	-0.004(-0.061,0.054)	-0.119	0.905
Superior outer	-0.052(-0.113,0.009)	-1.673	0.094

Univariate Logistic Regression Analysis of COPD and Retinal Thickness

	Thining				Thickening			
Ref：No	β	χ^2	p	OR(95%CI)	β	χ^2	p	OR(95%CI)
Central	-0.137	0.258	0.611	0.872(0.515,1.477)	-0.002	0.000	0.994	0.998(0.613,1.624)
Temporal inner	-0.071	0.098	0.754	0.931(0.596,1.454)	0.026	0.006	0.937	1.027(0.532,1.981)

Inferior inner	0.129	0.283	0.595	1.138(0.706,1.834)	0.311	0.841	0.359	1.365(0.702,2.652)
Nasal inner	0.008	0.001	0.971	1.008(0.646,1.574)	0.348	0.779	0.378	1.416(0.654,3.066)
Superior inner	0.058	0.072	0.788	1.060(0.693,1.622)	0.620	3.194	0.074	1.860(0.942,3.673)
Temporal outer	0.220	0.961	0.327	1.246(0.803,1.933)	0.115	0.136	0.712	1.122(0.609,2.069)
Inferior outer	0.610	6.326	0.012*	1.840(1.144,2.960)	-0.152	0.277	0.599	0.859(0.487,1.514)
Nasal outer	0.110	0.167	0.682	1.116(0.659,1.891)	0.103	0.087	0.768	1.109(0.559,2.200)
Superior outer	-0.276	1.359	0.244	0.759(0.477,1.207)	0.122	0.131	0.718	1.130(0.583,2.188)

Univariate Logistic Regression Analysis of Sex and Retinal Thickness

	Thining				Thickening			
Ref：Male	β	χ^2	p	OR(95%CI)	β	χ^2	p	OR(95%CI)
Central	-0.260	5.502	0.019*	0.771(0.621,0.958)	0.182	2.941	0.086	1.199(0.974,1.477)
Temporal inner	-0.146	2.304	0.129	0.864(0.716,1.043)	-0.038	0.067	0.786	0.963(0.721,1.285)
Inferior inner	-0.377	12.932	<0.001*	0.686(0.559,0.842)	0.024	0.023	0.881	1.024(0.748,1.402)
Nasal inner	-0.278	8.422	0.004*	0.757(0.628,0.914)	-0.078	0.167	0.683	0.925(0,635,1.347)
Superior inner	-0.033	0.131	0.718	0.968(0.809,1.157)	0.405	5.019	0.025*	1.499(1.054,2.137)
Temporal outer	0.155	2.333	0.127	1.167(0.957,1.423)	0.303	5.269	0.022*	1.354(1.045,1.755)
Inferior outer	-0.288	5.559	0.018*	0.750(0.590,0.953)	0.082	0.548	0.459	1.086(0.873,1.350)
Nasal outer	-0.287	5.710	0.017*	0.750(0.593,0.950)	0.069	0.214	0.644	1.071(0.801,1.433)
Superior outer	0.054	0.317	0.573	1.056(0.874,1.275)	0.102	0.536	0.464	1.107(0.843,1.453)

Univariate Logistic Regression Analysis of Age (Categorical Variable) and Retinal Thickness

Ref：<60 years		Thining β	χ²	p	OR(95%CI)	Thickening β	χ²	p	OR(95%CI)
Central	60-69	0.719	2.324	0.127	2.052(0.814,5.172)	-0.170	0.311	0.577	0.843(0.464,1.534)
	70-79	0.917	3.772	0.052	2.502(0.992,6.314)	0.016	0.003	0.959	1.016(0.558,1.851)
	＞80	1.096	4.739	0.029*	2.991(1.115,8.023)	0.096	0.074	0.785	1.101(0.552,2.194)
Temporal inner	60-69	0.596	2.178	0.140	1.815(0.822,4.004)	-0.193	0.222	0.637	0.824(0.369,1.842)
	70-79	1.109	7.564	0.006*	3.030(1.375,6.677)	-0.072	0.030	0.862	0.931(0.414,2.092)
	＞80	1.210	7.908	0.005*	3.352(1.443,7.788)	0.026	0.003	0.957	1.026(0.400,2.634)
Inferior inner	60-69	0.761	2.631	0.105*	2.140(0.853,5.368)	0.228	0.187	0.665	1.256(0.448,3.518)
	70-79	1.202	6.582	0.010*	3.327(1.328,8.334)	0.293	0.307	0.580	1.340(0.476,3.777)
	＞80	1.288	6.730	0.009*	3.626(1.370,9.596)	0.582	1.005	0.316	1.789(0.574,5.581)
Nasal inne	60-69	0.494	1.690	0.194	1.639(0.778,3.454)	-0.168	0.100	0.752	0.845(0.298,2.395)
	70-79	0.929	5.983	0.014*	2.532(1.203,5.33)	-0.205	0.145	0.703	0.815(0.284,2.338)
	＞80	0.994	5.887	0.015*	2.701(1.210,6.028)	0.256	0.181	0.670	1.292(0.397,4.201)
Superior inner	60-69	-0.059	0.039	0.843	0.943(0.528,1.684)	-0.155	0.105	0.746	0.856(0.335,2.191)
	70-79	0.318	1.158	0.282	1.375(0.770,2.454)	-0.314	0.417	0.518	0.730(0.281,1.896)
	＞80	0.457	1.932	0.165	1.579(0.829,3.006)	-0.506	0.700	0.403	0.603(0.184,1.973)
Temporal outer	60-69	-0.431	1.957	0.162	0.650(0.355,1.189)	-0.238	0.455	0.500	0.788(0.395,1.574)
	70-79	0.191	0.389	0.533	1.211(0.664,2.206)	-0.444	1.529	0.216	0.641(0.317,1.297)
	＞80	0.116	0.112	0.737	1.123(0.571,2.207)	-1.088	5.045	0.025*	0.337(0.13,0.871)
	60-69	1.222	2.849	0.091	3.396(0.821,14.040)	-0.487	2.804	0.094	0.615(0.348,1.086)

Inferior outer	70-79	1.694	5.483	0.019*	5.441(1.318,22.465)	-0.291	0.991	0.320	0.748(0.422,1.325)
	＞80	1.674	4.999	0.025*	5.333(1.23,23.135)	-0.486	1.868	0.172	0.615(0.307,1.235)
	60-69	0.292	0.451	0.502	1.339(0.571,3.139)	-0.098	0.057	0.811	0.907(0.407,2.021)
Nasal outer	70-79	0.485	1.244	0.265	1.625(0.692,3.812)	-0.306	0.546	0.460	0.736(0.327,1.659)
	＞80	0.456	0.908	0.341	1.577(0.618,4.025)	-0.931	2.831	0.092	0.394(0.133,1.166)
	60-69	-0.607	4.625	0.032*	0.545(0.313,0.948)	-0.432	1.476	0.224	0.649(0.323,1.304)
Superior outer	70-79	-0.024	0.007	0.933	0.976(0.563,1.693)	-0.716	3.866	0.049*	0.489(0.239,0.998)
	＞80	-0.048	0.023	0.879	0.953(0.509,1.782)	-0.913	3.855	0.050*	0.401(0.161,0.998)

Univariate Logistic Regression Analysis of History of Drinking and Retinal Thickness

	Thining				Thickening			
Ref：No	β	χ^2	p	OR(95%CI)	β	χ^2	p	OR(95%CI)
Central	0.062	0.164	0.685	1.064(0.787,1.440)	-0.341	4.289	0.038*	0.711(0.515,0.982)
Temporal inner	-0.013	0.009	0.926	0.987(0.752,1.295)	0.100	0.241	0.623	1.106(0.741,1.650)
Inferior inner	0.057	0.150	0.699	1.059(0.793,1.414)	-0.157	0.427	0.514	0.855(0.534,1.369)
Nasal inner	0.081	0.362	0.547	1.084(0.833,1.412)	-0.244	0.662	0.416	0.783(0.435,1.411)
Superior inner	-0.034	0.067	0.795	0.967(0.748,1.250)	-0.274	1.009	0.315	0.761(0.446,1.297)
Temporal outer	-0.286	3.498	0.061	0.751(0.557,1.014)	-0.383	3.477	0.062	0.682(0.456,1.020)
Inferior outer	-0.097	0.290	0.590	0.908(0.638,1.291)	-0.073	0.205	0.651	0.930(0.679,1.274)
Nasal outer	0.267	2.768	0.096	1.306(0.954,1.790)	-0.072	0.108	0.743	0.931(0.607,1.428)
Superior outer	-0.142	0.998	0.318	0.868(0.658,1.146)	-0.030	0.023	0.879	0.970(0.659,1.429)

Univariate Logistic Regression Analysis of History of Hypertension and Retinal Thickness

	Thining				Thickening			
Ref：No	β	χ^2	p	OR(95%CI)	β	χ^2	p	OR(95%CI)
Central	0.131	1.406	0.236	1.140(0.918,1.417)	0.028	0.073	0.787	1.029(0.839,1.261)
Temporal inner	0.227	5.556	0.018*	1.254(1.039,1.514)	0.215	2.144	0.143	1.240(0.930,1.654)
Inferior inner	0.053	0.253	0.615	1.054(0.859,1.294)	0.182	1.311	0.252	1.199(0.879,1.637)
Nasal inner	0.110	1.331	0.249	1.117(0.926,1.347)	0.123	0.415	0.519	1.131(0.778,1.645)
Superior inner	0.053	0.334	0.563	1.054(0.882,1.260)	0.023	0.018	0.892	1.024(0.729,1.437)
Temporal outer	0.127	1.606	0.205	1.135(0.933,1.382)	0.003	0.000	0.983	1.003(0.779,1.291)
Inferior outer	0.195	2.536	0.111	1.215(0.956,1.545)	0.042	0.145	0.703	1.043(0.841,1.294)
Nasal outer	0.205	2.888	0.089	1.227(0.969,1.554)	-0.018	0.015	0.902	0.982(0.737,1.309)
Superior outer	0.248	6.672	0.010*	1.281(1.062,1.546)	-0.226	2.671	0.102	0.798(0.608,1.046)

Univariate Logistic Regression Analysis of History of Diabetes and Retinal Thickness

	Thining				Thickening			
Ref：No	β	χ^2	p	OR(95%CI)	β	χ^2	p	OR(95%CI)
Central	0.025	0.038	0.845	1.025(0.800,1.314)	0.043	0.132	0.717	1.044(0.827,1.318)
Temporal inner	-0.144	1.637	0.201	0.866(0.695,1.079)	-0.190	1.181	0.277	0.827(0.588,1.165)
Inferior inner	-0.095	0.604	0.437	0.910(0.716,1.155)	-0.097	0.275	0.600	0.908(0.631,1.305)
Nasal inner	-0.212	3.503	0.061	0.809(0.647,1.010)	-0.251	1.183	0.277	0.778(0.495,1.223)
Superior inner	-0.124	1.369	0.242	0.883(0.717,1.088)	0.008	0.002	0.968	1.008(0.685,1.484)

Temporal outer	-0.332	7.418	0.006*	0.717(0.565,0.911)	-0.048	0.107	0.743	0.953(0.713,1.272)
Inferior outer	-0.170	1.367	0.242	0.844(0.635,1.122)	-0.026	0.041	0.840	0.975(0.761,1.249)
Nasal outer	-0.378	6.364	0.012*	0.685(0.511,0.919)	0.014	0.007	0.935	1.014(0.731,1.405)
Superior outer	-0.277	5.854	0.016*	0.758(0.605,0.949)	-0.121	0.570	0.450	0.886(0.648,1.212)

Univariate Logistic Regression Analysis of History of Coronary heart disease and Retinal Thickness

	Thining				Thickening			
Ref：No	β	χ^2	p	OR (95%CI)	β	χ^2	p	OR(95%CI)
Central	-0.029	0.032	0.857	0.972(0.709,1.330)	0.273	3.869	0.049*	1.314(1.001,1.724)
Temporal inner	0.000	0.000	0.999	1.000(0.765,1.306)	0.212	1.180	0.277	1.236(0.843,1.813)
Inferior inner	-0.037	0.060	0.806	0.964(0.719,1.293)	0.183	0.743	0.389	1.200(0.792,1.819)
Nasal inner	-0.083	0.353	0.552	0.921(0.701,1.209)	0.492	4.441	0.035*	1.636(1.035,2.586)
Superior inner	0.069	0.287	0.592	1.071(0.833,1.376)	0.397	3.275	0.070	1.488(0.968,2.288)
Temporal outer	0.075	0.287	0.592	1.078(0.818,1.421)	0.329	3.833	0.050*	1.390(1.000,1.932)
Inferior outer	0.245	2.227	0.136	1.277(0.926,1.761)	0.223	2.237	0.135	1.249(0.933,1.672)
Nasal outer	0.028	0.029	0.866	1.029(0.740,1.431)	-0.101	0.224	0.636	0.904(0.593,1.376)
Superior outer	-0.013	0.009	0.926	0.987(0.757,1.289)	0.093	0.243	0.622	1.098(0.758,1.590)

Univariate Logistic Regression Analysis of History of Hyperlipidemia and Retinal Thickness

	Thining				Thickening			
Ref：No	β	χ^2	p	OR (95%CI)	β	χ^2	p	OR (95%CI)
Central	-0.091	0.471	0.492	0.913(0.705,1.183)	0.167	2.017	0.156	1.182(0.939,1.488)

Temporal inner	0.076	0.479	0.489	1.079(0.871,1.336)	-0.531	7.294	0.007*	0.588(0.400,0.865)
Inferior inner	0.050	0.174	0.677	1.051(0.831,1.330)	-0.354	3.074	0.080	0.702(0.473,1.043)
Nasal inner	0.078	0.504	0.478	1.081(0.872,1.339)	-0.371	2.275	0.132	0.690(0.426,1.118)
Superior inner	0.129	1.555	0.212	1.138(0.929,1.395)	-0.085	0.168	0.681	0.919(0.612,1.378)
Temporal outer	-0.053	0.204	0.652	0.949(0.754,1.193)	-0.22	1.958	0.162	0.803(0.590,1.092)
Inferior outer	0.074	0.283	0.595	1.077(0.819,1.416)	-0.102	0.612	0.434	0.903(0.699,1.166)
Nasal outer	0.128	0.886	0.347	1.136(0.871,1.483)	-0.141	0.637	0.425	0.868(0.614,1.228)
Superior outer	0.080	0.539	0.463	1.083(0.875,1.342)	-0.236	1.948	0.163	0.790(0.567,1.100)

Univariate Logistic Regression Analysis of Moderate-intensity exercise over 30 mins and Retinal Thickness

		Thining				Thickening			
Ref：0 Time/week		β	χ^2	p	OR (95%CI)	β	χ^2	p	OR (95%CI)
Central	1-2	-0.095	0.307	0.579	0.910(0.651,1.272)	-0.272	2.795	0.095	0.762(0.553,1.048)
	3-4	-0.218	0.889	0.346	0.804(0.511,1.265)	-0.193	0.833	0.361	0.824(0.545,1.248)
	5-6	-0.233	0.799	0.372	0.792(0.476,1.32)	-0.132	0.324	0.569	0.876(0.555,1.382)
	≥7	-0.333	4.652	0.031*	0.716(0.529,0.97)	-0.382	7.089	0.008*	0.683(0.515,0.904)
Temporal inner	1-2	-0.107	0.509	0.475	0.898(0.669,1.206)	0.046	0.038	0.845	1.047(0.659,1.665)
	3-4	-0.386	3.321	0.068	0.680(0.449,1.030)	0.213	0.541	0.462	1.237(0.702,2.181)
	5-6	-0.202	0.820	0.365	0.817(0.527,1.266)	-0.383	0.971	0.325	0.682(0.318,1.461)
	≥7	-0.174	1.706	0.191	0.840(0.647,1.091)	-0.042	0.039	0.844	0.959(0.633,1.454)
Inferior inner	1-2	-0.117	0.499	0.480	0.889(0.642,1.232)	-0.112	0.207	0.649	0.894(0.551,1.449)
	3-4	-0.386	2.724	0.099	0.680(0.430,1.075)	-0.223	0.452	0.501	0.800(0.418,1.533)
	5-6	-0.078	0.105	0.746	0.925(0.577,1.482)	-0.322	0.683	0.409	0.725(0.338,1.555)

	≥7	-0.099	0.464	0.496	0.905(0.680,1.205)	-0.181	0.684	0.408	0.834(0.543,1.282)
Nasal inner	1-2	-0.052	0.118	0.731	0.950(0.707,1.276)	-0.378	1.653	0.199	0.685(0.385,1.219)
	3-4	-0.410	3.685	0.055	0.664(0.437,1.009)	-0.440	1.222	0.269	0.644(0.295,1.405)
	5-6	-0.259	1.260	0.262	0.772(0.492,1.213)	-0.358	0.667	0.414	0.699(0.296,1.651)
	≥7	-0.120	0.797	0.372	0.887(0.682,1.154)	-0.365	2.097	0.148	0.694(0.423,1.138)
Superior inner	1-2	-0.138	0.929	0.335	0.871(0.658,1.153)	0.032	0.011	0.916	1.032(0.575,1.854)
	3-4	-0.334	2.880	0.090	0.716(0.487,1.053)	0.256	0.507	0.477	1.292(0.638,2.614)
	5-6	0.094	0.221	0.638	1.099(0.742,1.628)	-0.052	0.013	0.908	0.949(0.392,2.297)
	≥7	-0.215	2.862	0.091	0.807(0.629,1.035)	0.225	0.749	0.387	1.252(0.752,2.085)
Temporal outer	1-2	0.104	0.405	0.524	1.110(0.805,1.529)	0.122	0.32	0.572	1.129(0.741,1.722)
	3-4	0.047	0.048	0.827	1.049(0.686,1.603)	0.393	2.286	0.131	1.481(0.89,2.465)
	5-6	0.097	0.169	0.681	1.101(0.695,1.745)	0.012	0.002	0.969	1.012(0.542,1.89)
	≥7	0.115	0.628	0.428	1.122(0.844,1.492)	0.160	0.697	0.404	1.173(0.806,1.707)
Inferior outer	1-2	0.115	0.323	0.570	1.122(0.754,1.669)	-0.226	1.531	0.216	0.798(0.558,1.141)
	3-4	0.158	0.363	0.547	1.171(0.701,1.956)	0.154	0.482	0.488	1.167(0.755,1.803)
	5-6	0.040	0.018	0.894	1.041(0.577,1.879)	0.225	0.869	0.351	1.252(0.780,2.010)
	≥7	0.174	0.929	0.335	1.190(0.835,1.696)	0.001	0.000	0.997	1.001(0.737,1.358)
Nasal outer	1-2	0.344	3.156	0.076	1.411(0.965,2.062)	-0.293	1.584	0.208	0.746(0.472,1.178)
	3-4	0.308	1.549	0.213	1.361(0.838,2.212)	0.010	0.001	0.973	1.010(0.575,1.773)
	5-6	0.314	1.327	0.249	1.369(0.802,2.337)	-0.043	0.017	0.896	0.958(0.507,1.812)
	≥7	-0.035	0.036	0.849	0.966(0.677,1.379)	-0.265	1.768	0.184	0.767(0.519,1.134)
Superior outer	1-2	-0.027	0.033	0.857	0.973(0.723,1.309)	0.161	0.469	0.494	1.175(0.740,1.866)
	3-4	0.030	0.022	0.881	1.030(0.699,1.517)	0.466	2.725	0.099	1.593(0.916,2.769)
	5-6	-0.027	0.015	0.902	0.974(0.636,1.49)	-0.737	2.615	0.106	0.479(0.196,1.169)
	≥7	-0.121	0.806	0.369	0.886(0.68,1.154)	0.311	2.240	0.134	1.365(0.908,2.052)

Univariate Logistic Regression Analysis of Weekly Intake of Fruits/Vegetables and Retinal Thickness

		Thining				Thickening			
Ref：Almost not		β	χ^2	p	OR (95%CI)	β	χ^2	p	OR (95%CI)
Central	＜1 kg	-0.004	0.000	0.991	0.996(0.505,1.967)	0.321	0.738	0.390	1.378(0.663,2.866)
	1-1.5 kg	-0.066	0.037	0.847	0.936(0.477,1.835)	0.222	0.357	0.550	1.248(0.603,2.584)
	1.5-2 kg	-0.616	2.759	0.097	0.540(0.261,1.117)	-0.018	0.002	0.962	0.982(0.46,2.094)
	≥2 kg	-0.018	0.003	0.958	0.982(0.496,1.943)	0.342	0.838	0.360	1.408(0.676,2.932)
Temporal inner	＜1 kg	-0.281	0.952	0.329	0.755(0.430,1.327)	0.279	0.206	0.650	1.322(0.396,4.406)
	1-1.5 kg	-0.358	1.576	0.209	0.699(0.400,1.222)	0.445	0.538	0.463	1.561(0.475,5.133)
	1.5-2 kg	-0.36	1.448	0.229	0.698(0.388,1.254)	0.576	0.864	0.353	1.779(0.528,5.995)
	≥2 kg	-0.321	1.226	0.268	0.726(0.411,1.280)	0.721	1.405	0.236	2.057(0.624,6.782)
Inferior inner	＜1 kg	0.126	0.135	0.713	1.134(0.580,2.216)	1.537	2.271	0.132	4.651(0.63,34.334)
	1-1.5 kg	-0.012	0.001	0.973	0.988(0.508,1.924)	1.386	1.851	0.174	3.997(0.543,29.42)
	1.5-2 kg	-0.156	0.192	0.661	0.855(0.425,1.721)	1.512	2.166	0.141	4.536(0.606,33.979)
	≥2 kg	0.152	0.197	0.657	1.164(0.595,2.279)	1.723	2.861	0.091	5.603(0.761,41.271)
Nasal inner	＜1 kg	0.047	0.023	0.880	1.048(0.569,1.930)	0.228	0.093	0.760	1.256(0.290,5.437)
	1-1.5 kg	-0.078	0.064	0.801	0.925(0.505,1.695)	0.293	0.157	0.692	1.341(0.315,5.713)
	1.5-2 kg	-0.253	0.604	0.437	0.777(0.411,1.469)	0.518	0.473	0.492	1.679(0.384,7.347)
	≥2 kg	0.115	0.137	0.711	1.122(0.609,2.068)	0.564	0.579	0.447	1.758(0.411,7.521)
Superior inner	＜1 kg	-0.228	0.656	0.418	0.796(0.459,1.382)	1.222	1.425	0.233	3.395(0.456,25.261)
	1-1.5 kg	-0.251	0.816	0.366	0.778(0.451,1.341)	1.194	1.369	0.242	3.302(0.446,24.418)
	1.5-2 kg	-0.272	0.873	0.350	0.762(0.43,1.348)	1.431	1.932	0.165	4.181(0.556,31.441)
	≥2 kg	-0.133	0.223	0.637	0.876(0.505,1.52)	1.339	1.711	0.191	3.815(0.513,28.369)
Temporal outer	＜1 kg	0.315	0.785	0.376	1.370(0.683,2.748)	0.307	0.403	0.526	1.359(0.527,3.509)
	1-1.5 kg	0.212	0.360	0.549	1.236(0.619,2.468)	0.312	0.423	0.516	1.366(0.534,3.496)

	1.5-2 kg	0.331	0.829	0.363	1.393(0.683,2.843)	0.452	0.843	0.359	1.571(0.599,4.124)
	≥2 kg	0.307	0.740	0.390	1.359(0.676,2.733)	0.520	1.162	0.281	1.681(0.654,4.324)
Inferior outer	＜1 kg	0.483	1.013	0.314	1.622(0.633,4.157)	0.494	1.254	0.263	1.640(0.690,3.896)
	1-1.5 kg	0.512	1.153	0.283	1.668(0.655,4.245)	0.565	1.663	0.197	1.759(0.746,4.149)
	1.5-2 kg	0.644	1.739	0.187	1.903(0.731,4.953)	0.688	2.359	0.125	1.990(0.827,4.790)
	≥2 kg	0.585	1.481	0.224	1.794(0.700,4.601)	0.710	2.598	0.107	2.034(0.858,4.821)
Nasal outer	＜1 kg	0.294	0.505	0.477	1.342(0.596,3.018)	0.623	1.051	0.305	1.864(0.567,6.133)
	1-1.5 kg	0.094	0.052	0.820	1.098(0.490,2.463)	0.410	0.456	0.499	1.506(0.459,4.941)
	1.5-2 kg	0.023	0.003	0.957	1.024(0.441,2.376)	0.688	1.247	0.264	1.990(0.595,6.662)
	≥2 kg	0.220	0.281	0.596	1.247(0.552,2.815)	0.681	1.257	0.262	1.976(0.601,6.504)
Superior outer	＜1 kg	0.126	0.155	0.694	1.135(0.605,2.127)	0.711	1.370	0.242	2.037(0.619,6.703)
	1-1.5 kg	0.001	0.000	0.998	1.001(0.536,1.869)	0.805	1.781	0.182	2.236(0.686,7.292)
	1.5-2 kg	0.294	0.808	0.369	1.342(0.706,2.550)	0.682	1.214	0.271	1.979(0.588,6.662)
	≥2 kg	0.194	0.364	0.546	1.214(0.647,2.277)	0.793	1.703	0.192	2.211(0.672,7.276)

Univariate Logistic Regression Analysis of Salty Taste Preference and Retinal Thickness

	Thining				Thickening			
Ref：No	β	χ^2	p	OR (95%CI)	β	χ^2	p	OR (95%CI)
Central	-0.204	1.617	0.204	0.816(0.596,1.117)	-0.113	0.591	0.442	0.893(0.67,1.191)
Temporal inner	0.041	0.097	0.756	1.0420(0.806,1.346)	-0.140	0.435	0.509	0.870(0.574,1.317)
Inferior inner	-0.006	0.002	0.968	0.994(0.749,1.320)	-0.027	0.014	0.904	0.974(0.632,1.500)
Nasal inner	0.096	0.555	0.456	1.101(0.855,1.418)	-0.192	0.459	0.498	0.826(0.474,1.437)
Superior inner	0.074	0.365	0.546	1.077(0.846,1.372)	-0.298	1.26	0.262	0.743(0.442,1.249)
Temporal outer	-0.080	0.336	0.562	0.923(0.703,1.211)	-0.558	7.011	0.008*	0.572(0.378,0.865)

Inferior outer	0.224	2.014	0.156	1.251(0.918,1.705)	-0.241	2.174	0.140	0.786(0.571,1.083)
Nasal outer	0.322	4.453	0.035*	1.380(1.023,1.860)	-0.187	0.735	0.391	0.83(0.542,1.271)
Superior outer	0.063	0.236	0.627	1.065(0.826,1.373)	-0.192	0.907	0.341	0.825(0.556,1.225)

Univariate Logistic Regression Analysis of Regular Consumption of Pickled Foods and Retinal Thickness

	Thining				Thickening			
Ref：No	β	χ^2	p	OR (95%CI)	β	χ^2	p	OR (95%CI)
Central	0.094	0.402	0.526	1.099(0.821,1.470)	0.076	0.292	0.589	1.079(0.819,1.421)
Temporal inner	-0.013	0.010	0.921	0.987(0.765,1.274)	-0.370	2.709	0.100	0.691(0.445,1.073)
Inferior inner	-0.138	0.879	0.348	0.871(0.653,1.162)	-0.229	0.987	0.321	0.795(0.506,1.250)
Nasal inner	-0.126	0.887	0.346	0.881(0.678,1.146)	-0.114	0.180	0.671	0.892(0.527,1.511)
Superior inner	0.137	1.297	0.255	1.147(0.906,1.453)	-0.315	1.410	0.235	0.730(0.434,1.227)
Temporal outer	0.111	0.693	0.405	1.117(0.860,1.451)	-0.041	0.052	0.819	0.960(0.675,1.364)
Inferior outer	0.041	0.060	0.806	1.042(0.752,1.443)	0.162	1.258	0.262	1.176(0.886,1.562)
Nasal outer	-0.321	3.195	0.074	0.725(0.510,1.031)	-0.073	0.131	0.718	0.929(0.625,1.383)
Superior outer	0.199	2.539	0.111	1.220(0.955,1.559)	-0.195	0.940	0.332	0.822(0.554,1.221)
