# Supplementary figures and images for: Analysis of factors influencing retinal thickness in chronic obstructive pulmonary disease and hypertension: a cross-sectional study in a community-dwelling middle-aged and elderly population
Source: Front Med (Lausanne). 2026 Mar 3;13:1752515. doi: 10.3389/fmed.2026.1752515 (PMC12994154; doi:10.3389/fmed.2026.1752515)

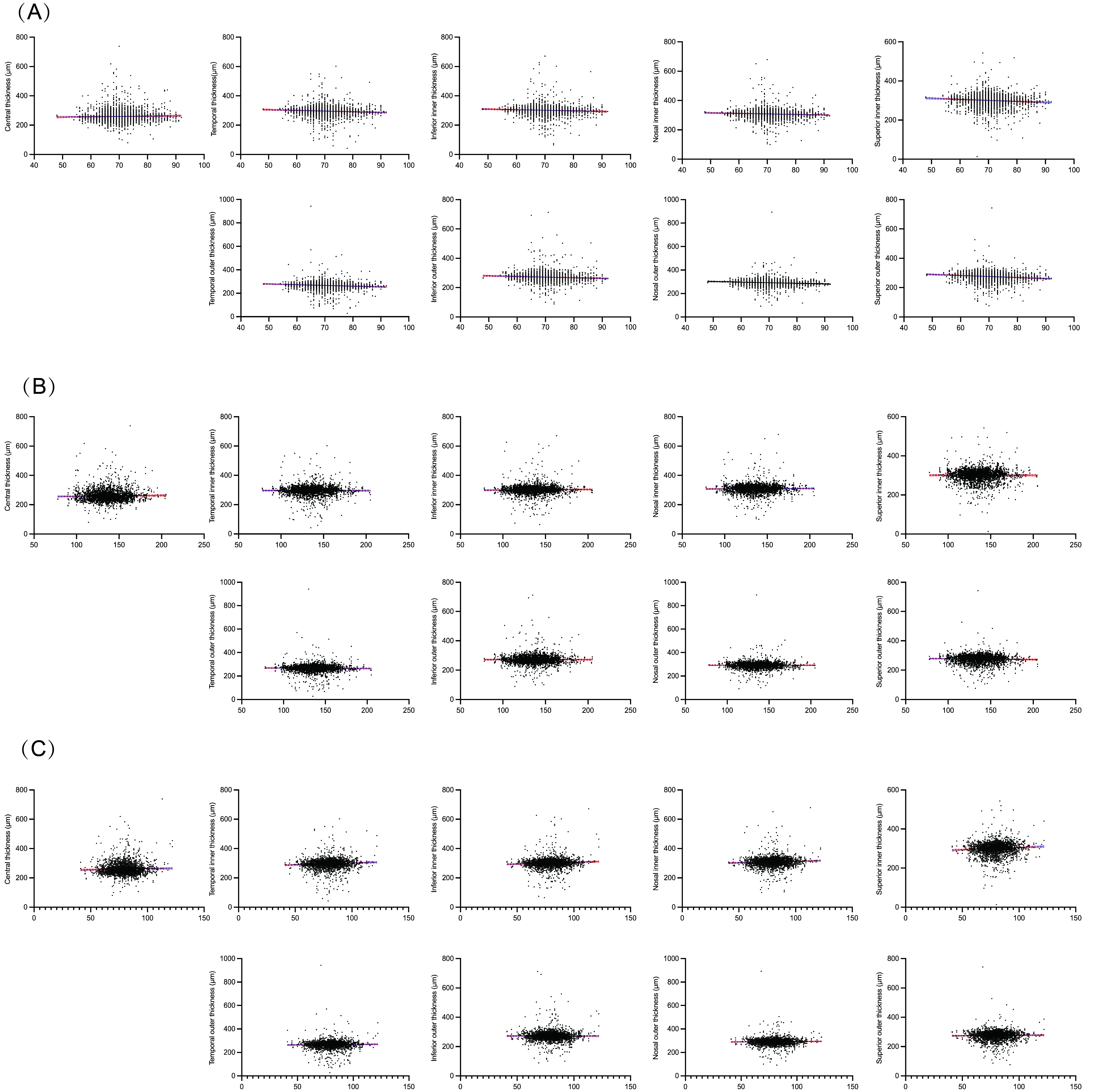

Supplement: Supplementary file 2 [file Image_1.JPEG]

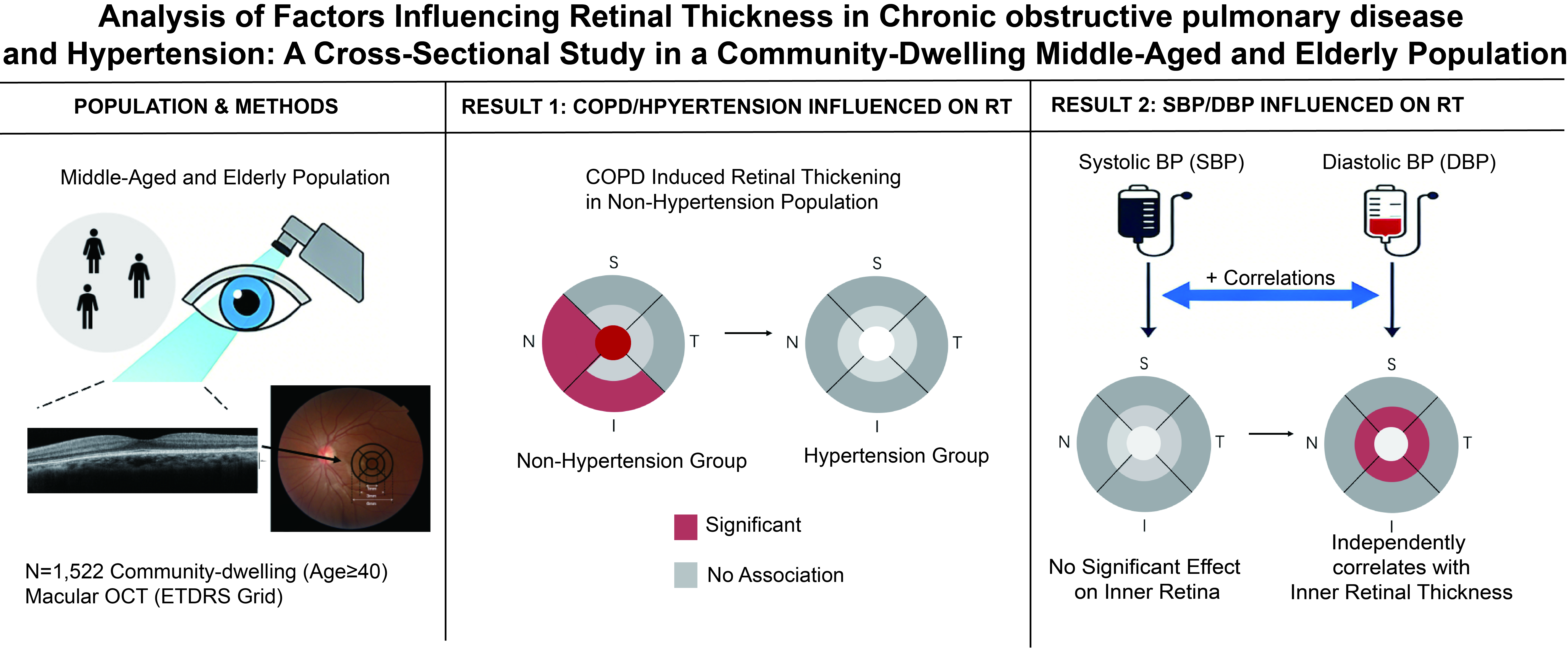

Supplement: Supplementary file 3 [file Image_2.JPEG]
